# Comparison of surface matching and target matching for image‐guided pelvic radiation therapy for both supine and prone patient positions

**DOI:** 10.1120/jacmp.v17i3.5611

**Published:** 2016-05-08

**Authors:** Hui Zhao, Brian Wang, Vikren Sarkar, Prema Rassiah‐Szegedi, Y. Jessica Huang, Martin Szegedi, Long Huang, Victor Gonzalez, Bill Salter

**Affiliations:** ^1^ Department of Radiation Oncology University of Utah Salt Lake City UT USA; ^2^ Department of Radiation Oncology University of Louisville Louisville KY USA; ^3^ Department of Radiation Oncology University of Arizona Cancer Center Tucson AZ USA

**Keywords:** AlignRT, CT‐on‐rails, IGRT, supine, prone

## Abstract

We investigate the difference between surface matching and target matching for pelvic radiation image guidance. The uniqueness of our study is that all patients have multiple CT‐on‐rails (CTOR) scans to compare to corresponding AlignRT images. Ten patients receiving pelvic radiation were enrolled in this study. Two simulation CT scans were performed in supine and prone positions for each patient. Body surface contours were generated in treatment planning system and exported to AlignRT to serve as reference images. During treatment day, the patient was aligned to treatment isocenter with room lasers, and then scanned with both CTOR and AlignRT. Image‐guidance shifts were calculated for both modalities by comparison to the simulation CT and the differences between them were analyzed for both supine and prone positions, respectively. These procedures were performed for each patient once per week for five weeks. The difference of patient displacement between AlignRT and CTOR was analyzed. For supine position, five patients had an average difference of displacement between AlignRT and CTOR along any direction (vertical, longitudinal, and lateral) greater than 0.5 cm, and one patient greater than 1 cm. Four patients had a maximum difference greater than 1 cm. For prone position, seven patients had an average difference greater than 0.5 cm, and three patients greater than 1 cm. Nine patients had a maximum difference greater than 1 cm. The difference of displacement between AlignRT and CTOR was greater for the prone position than for the supine position. For the patients studied here, surface matching does not appear to be an advisable image‐guidance approach for pelvic radiation therapy for patients with either supine or prone position. There appears to be a potential for large alignment discrepancies (up to 2.25 cm) between surface matching and target matching.

PACS number(s): 87.55.‐x

## I. INTRODUCTION

Accurate target localization and verification for pelvic radiation therapy prior to IMRT delivery is essential, due to the interfractional change of target position that is common in the pelvis second to day‐to‐day variations of bladder, bowel, and rectal filling. There are numerous imaging modalities available for image‐guided pelvic radiation therapy, including MV and kV portal imaging, MV and kV cone‐beam CT, in‐room CT‐on‐rails (CTOR), ultrasound, and Calypso (Varian Medical Systems, Palo Alto, CA) RF tracking (for prostate localization). Most of these modalities entail the delivery of extra radiation dose to patients. Ultrasound guidance may suffer from user subjective interpretation and Calypso requires invasive implantation of beacons.

AlignRT (Vision RT, London, UK) is a 3D surface imaging system for patient localizing, tracking, and monitoring during radiation therapy.^1^, ^2^ The advantages of the AlignRT system are its provision of real‐time tracking of patient surface location by monitoring of a region of interest during radiation therapy, its noninvasive imaging methodology, and its delivery of no extra radiation dose. It has been reported by multiple studies^3^, ^4^, ^5^, ^6^, ^7^, ^8^, ^9^, ^10^ that AlignRT can be an effective image guidance method for certain sites of radiation therapy treatment, including breast, intracranial stereotactic radiosurgery, thorax, and prostate.

However, it remains a question as to whether or not AlignRT surface matching can be effectively used for image‐guided pelvic radiation therapy. In this study, we investigate the difference between surface matching and target matching for pelvic radiation image guidance by comparing AlignRT with in‐room CT‐on‐rails. The uniqueness of our study is that all patients have multiple, same‐day CTOR scans to compare to the corresponding AlignRT 3D surface images. Our findings will be useful in providing guidance for future decisions concerning use of surface matching for image‐guided pelvic radiation therapy.

## II. MATERIALS AND METHODS

Ten patients with gynecologic or gastrointestinal malignancies were enrolled and analyzed in this study. Data acquisition and patient treatment were conducted according to institutional review board (IRB) protocol #39913. The median age was 60 (range 28–85), median height was 165.6 cm (range 150.0–185.0 cm), and median weight was 153 lbs (range 99–211 lbs). Table 1 shows the treatment position, height, and weight information for these patients.

At our center, the AlignRT system is installed in a vault that includes a Siemens Artiste linear accelerator and a Somatom Sensation 40 slice CTOR scanner (Siemens Healthcare, Erlangen, Germany). The AlignRT system is a two‐camera system (non‐HD cameras), and software version is 4.5. The two cameras are mounted on the ceiling, and the CTOR is oriented at 90° to the linac treatment couch.

The standard work flow for image‐guided radiation therapy using CTOR at our center is to align the patient with linac isocenter lasers first, then rotate the couch 90° to align the couch for CTOR image acquisition. After CTOR scan, the patient is rotated back to treatment position. Image fusion between treatment CTOR and simulation CT is performed and the offset between the two image sets is calculated. The patient is then shifted in three dimensions based on the CTOR‐derived fusion offset before treatment.

**Table 1:** Patient information in this study.

*Patient Number*	*Treatment Position*	*Age*	*Height (cm)*	*Weight (lbs)*
1	Supine	53	160.0	131
2	Prone	85	160.5	119
3	Supine	28	159.5	168
4	Supine	59	151.0	99
5	Prone	64	181.0	160
6	Prone	76	185.0	175
7	Supine	66	150.0	147
8	Supine	47	162.0	122
9	Supine	68	165.5	194
10	Prone	50	181.0	211

**Figure 1:**
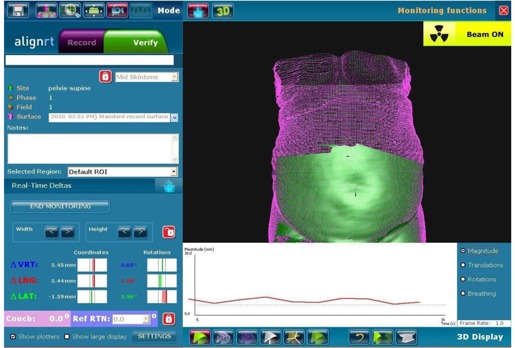
Snapshots of AlignRT real‐time surface tracking for supine position setup. The purple images were body surface contours automatically generated from corresponding simulation CT images. The green images were the real‐time tracking data, which were superimposed on the CT body contours to show the level of agreement. The range of the green image was preset as region of interest.

The image‐guidance workflow for this study entailed two initial simulation CT scans performed in supine and prone positions for each patient on a LightSpeed RT CT scanner (GE Healthcare, Waukesha, WI). In the supine position, patients were immobilized in alpha cradles; for the prone position, patients were immobilized in prone belly boards (Radiation Products Design, Albertville, MN). After the two CT datasets were imported into the treatment planning system, body surface contours were generated by the Eclipse treatment planning system (Varian Medical Systems, Palo Alto, CA) and exported to the AlignRT in‐room system to serve as reference images, according to the normal AlignRT workflow. The region of interest (ROI) was defined over the pelvic region without leg involvement to eliminate day‐to‐day leg position variation, and the side of pelvis above the alpha cradle or the prone belly board was also included for more accurate vertical alignment. It is noted that two separate CTs and treatment plans were acquired/produced for each patient — one for the supine and one for the prone position. In the treatment vault, once patients were aligned at their simulation‐defined treatment positions using linac isocenter lasers (i.e., at tattoos), AlignRT was initiated to record continuous patient displacement tracking for 1.5 to 2 min. The AlignRT topographic surface was compared with the body surface contour from the initial simulation CT within ROI, and the AlignRT shifts were calculated by averaging the tracking record (Fig. 1). Note that these AlignRT shifts were calculated during post‐treatment data analyses but not performed during treatment. The reason for calculating the shifts by averaging the tracking record is to eliminate the inconsistency caused by breathing movement. After AlignRT continuous tracking was recorded, patients were then scanned with the in‐room CTOR scanner, per our typical patient workflow. CTOR‐based image‐guidance shifts were derived by registering the CTOR dataset to the relevant (prone or supine) initial simulation CT image set. The registration was performed based on treatment target (GTV) alignment. With physician's approval, these image‐guidance shifts were applied. Since both sets of image‐guidance shifts were calculated from the initial setup position, the difference between calculated AlignRT shifts and CTOR shifts represent the difference between the two positioning systems' image‐guided solutions.

After treatment, patients were then set up in the alternate position orientation (i.e., supine if treated in the prone position, prone if treated in the supine position). AlignRT and CTOR image‐guidance shifts were again determined (as previously described) and the difference between the two systems' image‐guidance solutions were again recorded for the alternative position orientation. This process of determining the difference between AlignRT and CTOR image‐guidance shifts for both supine and prone patient orientations was performed for each of the 10 patients, once per week, for five weeks. This resulted in five “weekly” supine comparisons and five “weekly” prone comparisons for each patient, leading to a total of 50 total supine comparisons and 50 total prone comparisons for this study.

AlignRT and CTOR shifts from patient's tattoo aligned with linac isocenter lasers were analyzed for all patients. The difference of image‐guidance shifts between AlignRT and CTOR were calculated for each week's comparison in vertical, longitudinal, and lateral directions for both supine and prone positions of each patient. The average and maximum differences of patient displacement between AlignRT and CTOR over the five‐week treatment were calculated for each patient in the three cardinal directions.

## III. RESULTS

**Figure 2:**
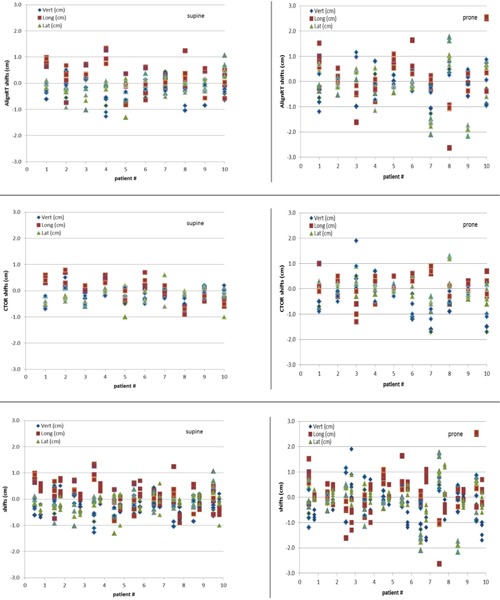
(Top row) AlignRT shifts along the vertical, longitudinal, and lateral directions from patient's tattoo aligned with linac isocenter lasers for all 10 patients over five‐week treatment. The figure on the left is for supine patient position, and the figure on the right is for prone patient position. (Middle row) CTOR shifts along the vertical, longitudinal, and lateral directions from patient's tattoo aligned with linac isocenter lasers. (Bottom row) AlignRT and CTOR shifts along the vertical, longitudinal, and lateral directions from patient's tattoo aligned with linac isocenter lasers. For each patient, the left group data are the AlignRT shifts, and the right group data are the CTOR shifts.

Figure 2 top row shows AlighRT shifts along the vertical, longitudinal, and lateral directions from patient's tattoo aligned with linac isocenter lasers for all 10 patients over five‐week treatment. The figure on the left is for supine patient position and the figure on the right is for prone patient position. The range of AlignRT translation shifts for supine position is up to 1.30 cm, and for prone position is up to 2.55 cm. The range of AlignRT rotation shifts for supine position is up to 2.14°, and for prone position is up to 9.69°. Figure 2 middle row shows CTOR shifts from patient's tattoo aligned with linac isocenter lasers. The figure on the left is for supine patient position and the figure on the right is for prone patient position. The range of CTOR translation shifts for supine position is up to 1.00 cm, and for prone position is up to 1.90 cm. Figure 2 bottom row shows both AlignRT and CTOR shifts in one graph for all patients. For each patient, the left group data are the AlignRT shifts and the right group data are the CTOR shifts.

Table 2 shows the frequency of patient shifts for both AlignRT and CT‐on‐rails from patient's tattoo aligned with linac isocenter lasers for all 10 patients over five‐week treatment. The shifts along any direction (vertical, longitudinal, and lateral) are divided into four groups: less than 3 mm, between 3 mm and 5 mm, between 5 mm and 10 mm, and over 10 mm. The frequency is calculated for both supine and prone positions. Even though the percentage of each category between AlignRT and CTOR has no significant difference, the shifts between AlignRT and CTOR for some patients are significantly different, as seen from Fig. 2 bottom row. The correlation coefficient of shifts between AlignRT and CTOR are 0.13, 0.32, and 0.47 along vertical, longitudinal, and lateral directions, respectively, which shows weak correlation along all three directions.

Table 3 shows the average (with standard deviation) and maximum difference of imageguidance shifts between AlignRT and CTOR along the vertical, longitudinal, and lateral directions for supine positions for all 10 patients over five‐week treatment (10 patients, with five comparisons each, resulting in a total of 50 supine comparisons). Table 4 shows the same information as in Table 3 for prone positions (again, 10 patients, with five comparisons each, resulting in a total of 50 prone comparisons).

As seen from Table 3 for the supine position, there were five patients who had an average difference between AlignRT and CTOR along any direction that was greater than 0.5 cm, and one of these five patients had more than a 1 cm average difference (Patient #8). Patient #4 had greater than 0.5 cm average differences for both vertical and longitudinal directions. Additionally, there were four patients who had a maximum difference of displacement between AlignRT and CTOR along any direction greater than 1 cm. Patient #10 also had maximum differences greater than 1 cm in both the longitudinal and lateral directions.

As seen from Table 4 for the prone position, there were seven patients who had an average difference between AlignRT and CTOR along any direction greater than 0.5 cm, and three of these seven patients had greater than a 1 cm average difference. Patient #6 had greater than 0.5 cm average difference for two directions and Patients #7, #8, and #10 had average differences greater than 0.5 cm in all three directions. Additionally, nine of the ten prone patients had maximum difference of displacement between AlignRT and CTOR along any direction greater than 1 cm (all but Patient #2). Two patients, #6 and #8, also had maximum differences greater than 1 cm in two directions, and an additional two patients, #7 and #10, had maximum differences in all three directions, thus making it clear that the difference between AlignRT shifts and CTOR shifts is greatest for the patient prone position. A two‐tailed paired *t*‐test showed differences between AlignRT and CTOR between patient supine and prone positions along vertical and lateral directions were statistically significant (p‐values of $7.66\times 10^{-5}$ and 0.0076, respectively).

**Table 2:** Frequency of patient shifts for both AlignRT and CT‐on‐rails from patient's tattoo aligned with linac isocenter lasers for all 10 patients over five‐week treatment. The shifts along any direction (vertical, longitudinal, and lateral) are divided into four groups: less than 3 mm, between 3 mm and 5 mm, between 5 mm and 10 mm, and over 10 mm. The frequency is calculated for both supine and prone positions.

*Shift Range (mm)*	*Supine*		*Prone*	
	*AlignRT*	*CTOR*	*AlignRT*	*CTOR*
<3	50.0%	48.0%	37.3%	39.4%
≤3<5	18.0%	28.7%	18.7%	21.3%
≤5<10	24.7%	22.0%	24.0%	26.0%
≥3	7.3%	1.3%	20.0%	13.3%

**Table 3:** Averages and maximum difference of patient displacement between AlignRT and CT‐on‐rails along vertical, longitudinal, and lateral directions for supine positions over five‐week treatment.

	*Supine*					
	*Vert. (cm)*		*Long. (cm)*		*Lat. (cm)*	
*Patient #*	*Ave.*	*Max.*	*Ave.*	*Max.*	*Ave.*	*Max.*
1	0.29±0.18	0.45	0.34±0.19	0.54	0.31±0.13	0.52
2	0.37±0.14	0.58	0.32±0.30	0.84	0.24±0.18	0.52
3	0.42±0.14	0.62	0.35±0.23	0.73	0.25±0.15	0.52
4	0.99±0.30	1.41	0.70±0.18	0.94	0.10±0.08	0.23
5	0.37±0.25	0.73	0.27±0.14	0.43	0.10±0.12	0.30
6	0.19±0.17	0.44	0.61±0.43	1.34	0.33±0.17	0.53
7	0.32±0.23	0.54	0.21±0.15	0.43	0.25±0.14	0.45
8	0.30±0.33	0.75	1.12±0.55	1.95	0.12±0.09	0.25
9	0.22±0.21	0.55	0.65±0.27	0.97	0.34±0.07	0.41
10	0.24±0.27	0.54	0.56±0.38	1.10	0.78±0.41	1.25
mean	0.37±0.23	0.66±0.28	0.51±0.27	0.93±0.46	0.28±0.20	0.50±0.29

**Table 4:** Averages and maximum difference of patient displacement between AlignRT and CT‐on‐rails along vertical, longitudinal, and lateral directions for prone positions over five‐week treatment.

	*Prone*					
	*Vert. (cm)*		*Long. (cm)*		*Lat. (cm)*	
*Patient #*	*Ave.*	*Max.*	*Ave.*	*Max.*	*Ave.*	*Max.*
1	0.19±0.16	0.39	0.76±0.41	1.42	0.26±0.16	0.42
2	0.24±0.24	0.55	0.44±0.18	0.65	0.23±0.14	0.42
3	0.39±0.45	0.99	0.59±0.34	1.01	0.37±0.31	0.94
4	0.15±0.14	0.36	0.37±0.16	0.61	0.49±0.39	1.14
5	0.16±0.14	0.35	0.43±0.40	1.09	0.23±0.12	0.36
6	0.96±0.24	1.30	0.61±0.77	1.62	0.28±0.25	0.61
7	0.66±0.24	1.03	0.78±0.29	1.23	1.13±0.12	1.27
8	0.97±0.24	1.37	0.96±0.69	2.03	0.52±0.23	0.84
9	0.39±0.21	0.67	0.26±0.09	0.36	1.65±0.18	1.96
10	1.02±0.73	1.97	1.10±0.90	2.25	0.84±0.41	1.27
mean	0.51±0.36	0.90±0.54	0.63±0.27	1.23±0.62	0.60±0.47	1.02±0.53

Table 5 shows the range of difference of patient displacement between AlignRT and CTOR along vertical, longitudinal, and lateral directions for both supine and prone positions over five‐week treatment. Six patients had ranges of difference between AlignRT and CTOR greater than 1.0 cm along any direction, and one patient had a range of difference greater than 1.0 cm along all three directions. The range of difference showed day‐to‐day alignment variations between AlignRT and CTOR.

**Table 5:** The range of difference of patient displacement between AlignRT and CT‐on‐rails along vertical, longitudinal, and lateral directions for both supine and prone positions over five‐week treatment.

	*Supine*			*Prone*		
*Patient #*	*Vert. (cm)*	*Long. (cm)*	*Lat. (cm)*	*Vert. (cm)*	*Long. (cm)*	*Lat. (cm)*
1	0.01‐0.45	0.07‐0.54	0.17‐0.52	0.02‐0.39	0.35‐1.42	0.04‐0.42
2	0.22‐0.58	0.12‐0.84	0.04‐0.52	0.04‐0.55	0.23‐0.65	0.15‐0.42
3	0.26‐0.62	0.15‐0.73	0.14‐0.52	0.01‐0.99	0.06‐1.01	0.16‐0.94
4	0.62‐1.41	0.44‐0.94	0.01‐0.23	0.01‐0.36	0.19‐0.61	0.12‐1.14
5	0.11‐0.73	0.09‐0.43	0.04‐0.30	0.01‐0.35	0.04‐1.09	0.11‐0.36
6	0.05‐0.44	0.22‐1.34	0.11‐0.53	0.73‐1.30	0.00‐1.62	0.05‐0.61
7	0.00‐0.54	0.02‐0.43	0.08‐0.45	0.43‐1.03	0.47‐1.23	0.99‐1.27
8	0.03‐0.75	0.47‐1.95	0.01‐0.25	0.76‐1.37	0.22‐2.03	0.28‐0.84
9	0.02‐0.55	0.36‐0.97	0.26‐0.41	0.16‐0.67	0.13‐0.36	1.48‐1.96
10	0.00‐0.54	0.18‐1.10	0.19‐1.25	0.05‐1.97	0.08‐2.25	0.25‐1.27

## IV. DISCUSSION

We observed large AlignRT and CTOR shifts from patient's tattoo aligned with linac isocenter lasers for both supine and prone positions. This is reasonable since the three tattoo body marks at pelvic area would not be able to accurately represent the whole pelvic surface position and the internal target position. The shifts for prone position are larger than for supine position for both AlignRT and CTOR. This is mainly due to the difficulty of setup reproducibility for the prone position on the belly board.

**Figure 3:**
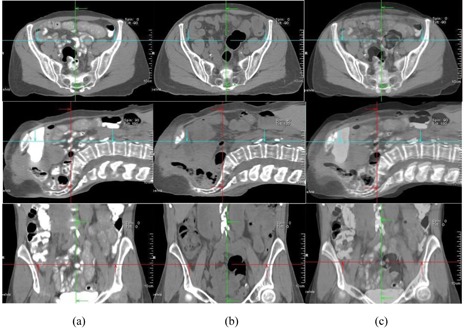
Comparison of simulation CT and CTOR after image registration for Patient #4 (supine), the fourth week of treatment. Images shown are the axial, sagittal, and frontal CT images for simulation CT (a), CTOR (b) and a blend view of simulation CT and CTOR after image registration (c).

**Figure 4:**
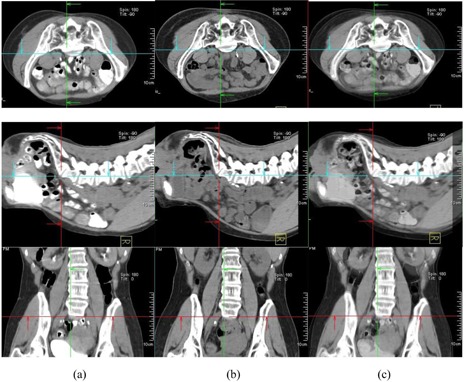
Comparison of simulation CT and CTOR after image registration for Patient #4 (prone), the fourth week of treatment. Images shown are the axial, sagittal, and frontal CT images for simulation CT (a), CTOR (b) and a blend view of simulation CT and CTOR after image registration (c).

In our study, large differences of patient displacement between AlignRT and CTOR were observed (up to 2.25 cm). This is not unexpected since, in our workflow, the registration between CTOR and simulation CT was based primarily on soft‐tissue target volumes (GTV) and adjacent bony anatomy; in contrast, patient shifts determined by AlignRT were based on patient skin surface alignment. The large differences of patient displacement between AlignRT and CTOR suggest large day‐to‐day variations of relative position between deep‐seated target volumes and patient surface. There are other reasons for the large difference, including different patient pelvic rotations, belly‐size and belly‐position changes at treatment days. 3, 6 illustrate these differences. Figure 3 shows a comparison of simulation CT and CTOR after image registration for Patient #4, the fourth week of treatment, in supine position. The relative position between patient skin surface and pelvic bony anatomy changed over 1.5 cm from simulation CT to the CTOR day. In addition to the skin surface position change, the shape of the patient's belly was very different between simulation CT and CTOR. Different pelvic tilts can also be distinguished from the fusion of simulation CT and CTOR. The green arrows on axial images and the red arrows on sagittal images show the same anterior/posterior level for simulation CT and CTOR. The bigger belly size in treatment day indicates possible weight gain and different bowel filling. Figure 4 shows a comparison of simulation CT and CTOR for the same patient (#4), on the same day of treatment as Fig. 3, in prone position. The bigger belly size on treatment day can also be observed in both axial and sagittal images for prone position. The spine/back curvature did not change much between simulation and treatment day, and neither did the relative position between target and patient back surface. Therefore, the difference between AlignRT and CTOR in prone position for this specific patient, on this specific treatment day, was not as large as in the supine position.

**Figure 5:**
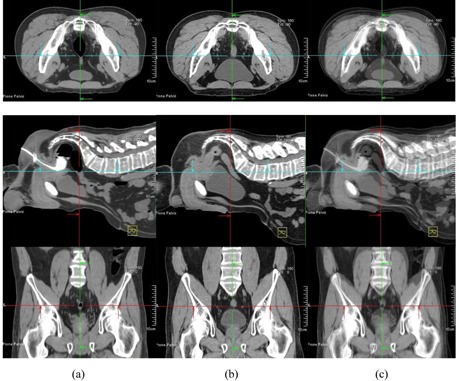
Comparison of simulation CT and CTOR after image registration for Patient #10 (prone), the third week of treatment. Images shown are the axial, sagittal, and frontal CT images for simulation CT (a), CTOR (b) and a blend view of simulation CT and CTOR after image registration (c).

**Figure 6:**
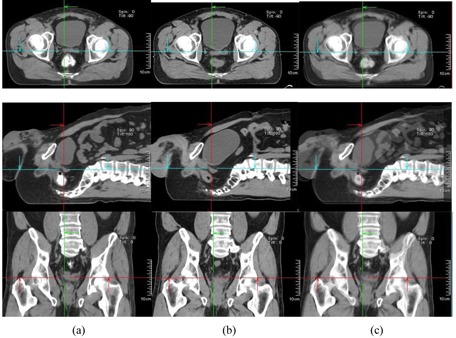
Comparison of simulation CT and CTOR after image registration for Patient #10 (supine), the third week of treatment. Images shown are the axial, sagittal, and frontal CT images for simulation CT (a), CTOR (b) and a blend view of simulation CT and CTOR after image registration (c).

Figure 5 shows a comparison of simulation CT and CTOR after image registration for Patient #10, the third week of treatment, in prone position. The relative position between patient skin surface and pelvic bony anatomy changed over 2.0 cm from simulation CT to the CTOR day. In addition to the skin surface position change, the curves of the patient's back were very different between simulation CT and CTOR (observed from sagittal images), as was the bladder filling. Figure 6 shows a comparison of simulation CT and CTOR for the same patient (#10), the same day of treatment as Fig. 5, in supine position. The bigger bladder filling on treatment day can be observed also in axial and sagittal images for supine position. The belly shape did not change much between simulation and treatment day, and neither did the relative position between target and patient belly surface. Therefore, the difference between AlignRT and CTOR in supine position for this specific patient, on this specific treatment day, was not as large as in the prone position. Different pelvic tilts could also be distinguished from the fusion of simulation CT and CTOR for both positions.

We identified another important factor affecting patient setup reproducibility, which is significant to pelvic radiation therapy, from this study. Consistent and accurate reproduction of patient setup for pelvic radiation therapy for both supine and prone positions can be extremely challenging. In addition to nonsetup‐related changes, such as bladder and bowel filling variations, and subsequent interfractional movement of the treatment target, we observed significant setup‐related variations such as patient pelvic rotation, pelvic tilt, and back shape changes. The inability to precisely reproduce the patient's simulation setup geometry can invalidate the relative skin to target geometric relationship that is required when using the skin surface as a surrogate for target position as is required for AlignRT guidance.

Additionally, we observed greater differences of patient displacement between AlignRT and CTOR for the patient prone position than for the patient supine position. This is primarily caused by greater day‐to‐day patient setup variations in patient prone position than in patient supine position. Most patients in our study had large bellies. Patients with large bellies are harder to consistently reposition for the prone setup orientation with prone belly board, than for the supine setup position in alpha cradles. The large day‐to‐day prone belly board position variation can cause a large change of relative position between GTV and patient surface.

In our study, we observed that the surface‐imaging approach to image guidance struggled to adapt to the common, day‐to‐day geometric variations in pelvic region anatomy for both patient supine and patient prone positions. On the contrary, CTOR's direct focus on treatment target (GTV) alignment eliminated the vulnerability associated with use of a skin surface surrogate for alignment and, thus, provided for more accurate target alignment.

To our knowledge, this study is the first study comparing AlignRT and CTOR for image‐guided pelvic radiation therapy. While there have been previously published studies of image‐guided prostate radiation therapy using AlignRT^9^, ^10^ these studies have primarily used EPID imaging for comparison, with emphasis placed on bony anatomy registration. Our study differs significantly from these studies in that we were able to exploit the superior image contrast of fan‐beam CT to allow for direct and accurate registration of the targeted soft tissue anatomy. Our acquisition of CTOR images of both supine and prone orientations, weekly for five weeks, for 10 different patients, has allowed for a thorough characterization of possible geometric challenges to using a skin surface surrogate for pelvic soft tissue targeting and, as such, should prove extremely valuable for assessing the viability of such an approach for targeting of pelvic lesions.

## V. CONCLUSIONS

For the patients studied here, the daily setup variations typical to pelvic RT patients, coupled with normal interfractional variations in relative soft tissue target location within the patient, rendered the skin‐surface surrogate approach of AlignRT to be potentially inaccurate for pelvic radiation therapy. We believe that for this specific application surface matching does not appear to be an advisable image‐guidance approach, as we saw potential for errors as large as 2.25 cm compared to target matching.

## COPYRIGHT

This work is licensed under a Creative Commons Attribution 4.0 International License.

## Supporting information

Supplementary Material
