# Supplementary material for: Comparison of surface matching and target matching for image‐guided pelvic radiation therapy for both supine and prone patient positions
Source: J Appl Clin Med Phys. 2016 May 8;17(3):14–24. doi: 10.1120/jacmp.v17i3.5611 (PMC5690902; doi:10.1120/jacmp.v17i3.5611)
Supplement: Supplementary file 1 — Supplementary Material [file ACM2-17-014-s001.docx]

Comparison of surface matching and target matching for image guided pelvic radiation therapy for both supine and prone patient positions

**Abstract:** Purpose: We investigate the difference between surface matching and target matching for pelvic radiation image guidance. The uniqueness of our study is that all patients have multiple CT-on-rails (CTOR) scans to compare to corresponding AlignRT images.

Methods/Materials: Ten patients receiving pelvic radiation were enrolled in this study. Two simulation CT scans were performed in supine and prone positions for each patient. Body surface contours were generated in treatment planning system and exported to AlignRT to serve as reference images. The patient was aligned to treatment isocenter with room lasers, and then scanned with both CTOR and AlignRT. Image guidance shifts were calculated for both modalities by comparison to the simulation CT and the differences between them were analyzed for both supine and prone positions, respectively. These procedures were performed for each patient once per week for five weeks. The difference of patient displacement between AlignRT and CTOR were analyzed.

Results: For supine position, there were 5 patients who had the average difference of displacement between AlignRT and CTOR along any direction (vertical, longitudinal, and lateral) greater than 0.5cm, and 1 patient greater than 1cm. There were 4 patients who had the maximum difference greater than 1cm. For prone position, there were 7 patients who had the average difference greater than 0.5cm, and 3 patients greater than 1cm. There were 9 patients who had the maximum difference greater than 1 cm. The difference of displacement between AlignRT and CTOR was greater for the prone position than for the supine position.

Conclusion: For the patients studied here, surface matching does not appear to be an advisable image guidance approach for pelvic radiation therapy for patients with either supine or prone position. There appears to be a potential for large alignment discrepancies (up to 2.25 cm) between surface matching and target matching.

Key words: AlignRT; CT-on-rails; IGRT; supine; prone

PACS number: 87.55.-x
